# Infrared pupillometry for outcome prediction after cardiac arrest and therapeutic hypothermia

**DOI:** 10.1186/cc12248

**Published:** 2013-03-19

**Authors:** T Suys, N Sala, AO Rossetti, M Oddo

**Affiliations:** 1 Lausanne University Hospital, Lausanne, Switzerland

## Introduction

Sedation and therapeutic hypothermia (TH) modify neurological examination and alter prognostic prediction of coma after cardiac arrest (CA). Additional tools, such as EEG and evoked potentials, improve prediction of outcome in this setting, but are not widely available and require significant implementation.

## Methods

Using a new device for infrared pupillometry, we examined the value of quantitative pupillary light reactivity (PLR) to predict outcome in comatose post-CA patients treated with TH. Twenty-four comatose CA patients treated with TH (33°C, 24 hours) were prospectively studied. The percentage variation in PLR was measured during TH (12 hours from CA), using the NeuroLight Algiscan^® ^(IDMED, Marseille, France). For each patient, three consecutive measures were performed and the best value was retained for analysis. The relationship of PLR with survival and neurological outcome (CPC scores) at 3 months was analyzed, and the predictive value of PLR was compared with that of standard clinical examination (motor response and brainstem reflexes) performed at 48 hours from CA.

## Results

Quantitative PLR was strongly associated with survival (median left-eye PLR 14% (11 to 16%) variation in survivors vs. 5.5% (4 to 8.5%) in nonsurvivors, *P <*0.0001) and 3-month neurological outcome (14% (11 to 21%) in patients with CPC 1 to 2 vs. 5.5% (4 to 8.5%) in those with CPC 3 to 5, *P <*0.0001). Comparable findings were obtained using right-eye PLR. A PLR >10% was 100% predictive of patient prognosis, with false-positive and false-negative rates of 0% for outcome. Clinical examination was significantly associated with outcome; however, motor response (MR) and brainstem reflexes (BRS) yielded higher false-positive and false-negative rates than PLR (Table 1).

## Conclusion

Quantitative PLR appears highly accurate and superior to standard neurological examination to predict outcome in patients with post-CA coma. Further study is warranted to confirm these promising findings.

**Table 1:** False-positive and false-negative rates for outcome (% of patients)

		Outcome				
					
		Good (CPC 1 to 2) (%)	Bad (CPC 3 to 5) (%)	*P *value	FNR (%)	FPR (%)
PLR						
	≤10%	0	100	<0.0001	0	0
	>10%	100	0			
BRS						
	Absent	0	33	0.02	0	28
	Present	39	28			
MR						
	Absent	11	50	0.02	11	11
	Present	28	11			

